# Data supporting the production of dietary fibers from sugarcane bagasse and sugarcane tops using microwave - assisted alkaline treatments

**DOI:** 10.1016/j.dib.2019.104026

**Published:** 2019-05-20

**Authors:** D.I. Llanes Gil-López, J.A. Lois-Correa, M.E. Sánchez-Pardo, M.A. Domínguez-Crespo, A.M. Torres-Huerta, A.E. Rodríguez-Salazar, V.N. Orta-Guzmán

**Affiliations:** a Instituto Politécnico Nacional, CICATA-Altamira. km 14.5 carretera Tampico–Puerto Industrial Altamira, Altamira, Tamps., CP 89600, Mexico; b Instituto Politécnico Nacional, ENCB, UPALM, Av. Wilfrido Massieu esq. Cda. Manuel Stampa s/n C.P. Gustavo A. Madero, Ciudad de Mexico, 07728, Mexico; c Instituto Politécnico Nacional, CICATA-*Querétaro*, Cerro Blanco No. 141 Col. Colinas del Cimatario, C.P. 76090, Querétaro, Qro, Mexico

## Abstract

The treatment of agroindustrial residues is an alternative to waste management and obtain products with added value. In this article, we describe the confocal microscopy images, the microbiological data, policosanol content and color measurement linked to the paper “production of dietary fibers from sugarcane bagasse and sugarcane tops using microwave - assisted alkaline treatments”. The data contain photographs after elaboration of noodles-type pasta and chapatti-type fermented bread; the confocal laser scanning micrographs, before and after including sugarcane bagasse and sugarcane tops fibers in foods. Microbiological analyses of total coliforms, molds and yeasts, and aerobic mesophiles were also presented according to Mexican Standard NOM- 247-SSA1-2008 which confirmed that the food is safe for human consumption. The data provided in this article have not been previously published and are available to enable critical or extended analyses.

**Table:** Specifications table

Subject area	*Sustainable agricultural residues*
More specific subject area	*Agroindustrial wastes*
Type of data	*Table, images, figures*
How data was acquired	Photographs were took after 30 min of processing foods, using a conventional camera. Confocal laser scanning microscopy was obtain using a Carl Zeiss LSM700. A MEMMERT UF 110 furnace and professional sharp carousel microwave (frequency of 2.45 GHz and power of 1100 W) were used to dehydrated the samples. Violet red bile agar (VRBA), potato dextrose agar (PDA) and standard methods agar (SMA). Color Reader (CR-10 Plus, Konica Minolta, Japan), Gas chromatograph with Flame Ionization Detector, FID (GC-17A, Shimadzu, Japan), automatic injector (AOC 20S), capillary DB-5 column.
Data format	*JPG, Raw and analyzed curves*
Experimental factors	For confocal laser scanning microscopy, markers (calcofluor and rhodamine B) were used to identify the fibers and carbohydrates. In the case of chapatti-type fermented bread, heparin (5000 units) was added before incorporating the markers. The scanning used excitations brought by the 405 nm emission and 514 nm emission lines of the He–Ne laser. Total coliforms, aerobic mesophiles, molds and yeasts were realized on dried samples 10 g sample/90 mL of nutrient solution, followed of different logarithmic dilutions from 10^−1^ to 10^−6^. Samples were storage in sterile bottle assays at 25 °C ± 1 for 120 h days (molds and yeasts) or 35 °C ± 2 (aerobic mesophiles) for 48 h or 35 °C ± 2 (Total coliforms) for 24 h. For policosanol (PC) analyses, individual standards were used for peak identification (Sigma Aldrich, >99% purity), tricosanol (C_23_), tetracosanol (C_24_), heptacosanol (C_27_) and octacosanol (C_28_); N-methyl-N-(trimethylsilyl) trifluoroacetamide (MSTFA, Sigma Aldrich) was the derivatization reagent.
Experimental features	Dispersion of added fibers on processing foods (pastas and fermented bread) was analyzed by confocal laser scanning microscopy. Microbiological tests (Total coliforms, aerobic mesophiles, molds and yeasts) were determined using plate method according to the Mexican Standard NOM- 247-SSA1-2008.
Data source location	CICATA-Altamira, IPN, Altamira, Tamaulipas, México. ENCB-IPN, Ciudad de México, México
Data accessibility	*Data are available in this article.*
Related research article	*D. I. Llanes Gil-López, J. A. Lois-Correa, M. E. Sánchez Pardo*_*,*_*M. A. Domínguez Crespo, A.M Torres Huerta, A.E. Rodríguez-Salazar, V. N. Orta-Guzmán*^*a*^*.* production of dietary fibers from sugarcane bagasse and sugarcane tops using microwave - assisted alkaline treatments. Industrial Crops and Products. *In press*.

**Table:** 

**Value of the data** • These data are valuable because show the food enrichment through agroindustrial waste fibers incorporation. • Data highlights conditions to reach a good integration of sugarcane bagasse and sugarcane tops fibers as ingredient of chapatti-type fermented bread and noodles-type pasta • Data show that sugarcane bagasse fibers can be used in fermented breads without detriment of fermentation features. • Data from this research can be used for new applications of agroindustrial wastes such as sugarcane bagasse and sugarcane tops fibers. • Data from this research demonstrate that microwave-assisted alkaline treatment enhances the integration of sugarcane bagasse and sugarcane tops fibers during the preparation of pasta (noodles) and bread (chapatti).

## 1 Data

It has been reported that the presence of dietary fiber in foods has beneficial effects in human health, such as prevent cardiac diseases, gastrointestinal problems, decrease cholesterol levels, among others [1], [2], [3], [4], [5]; the recommended quantity of fiber ingestion is ranged from 30 to 45 g per day [6], [7], [8], [9]. Then, adding fibers from agricultural wastes is a good choice to enhance foods, especially pasta and bread, and become into functional food rich in fiber.

**Fig. 1:**
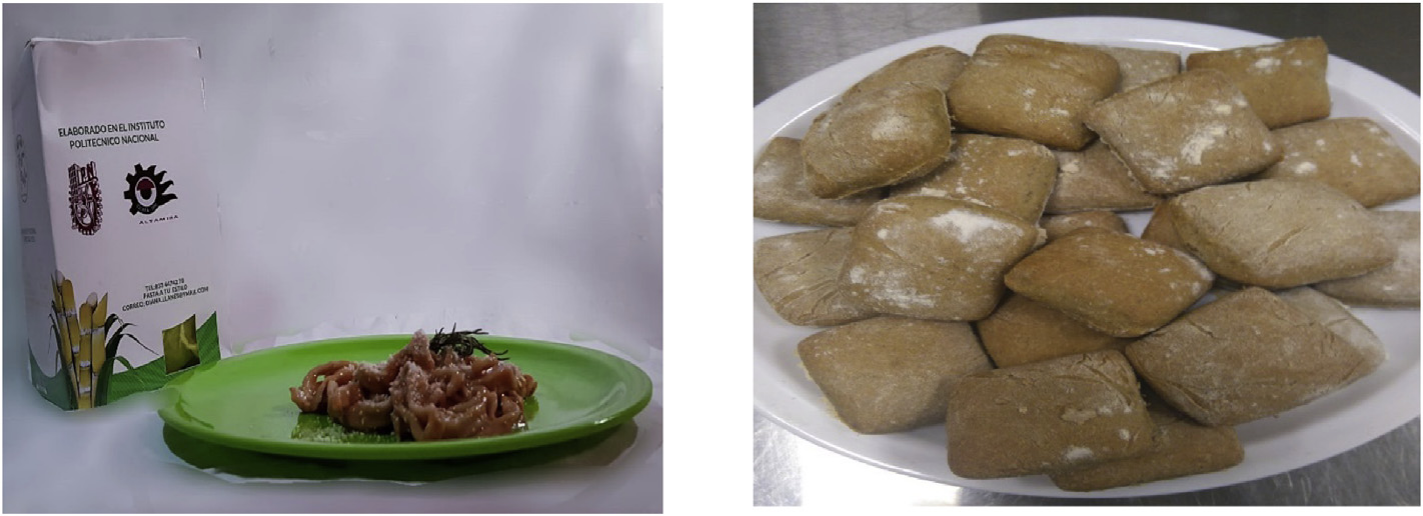
Photographs of noodles (left) and chapatti (right).

**Fig. 2:**
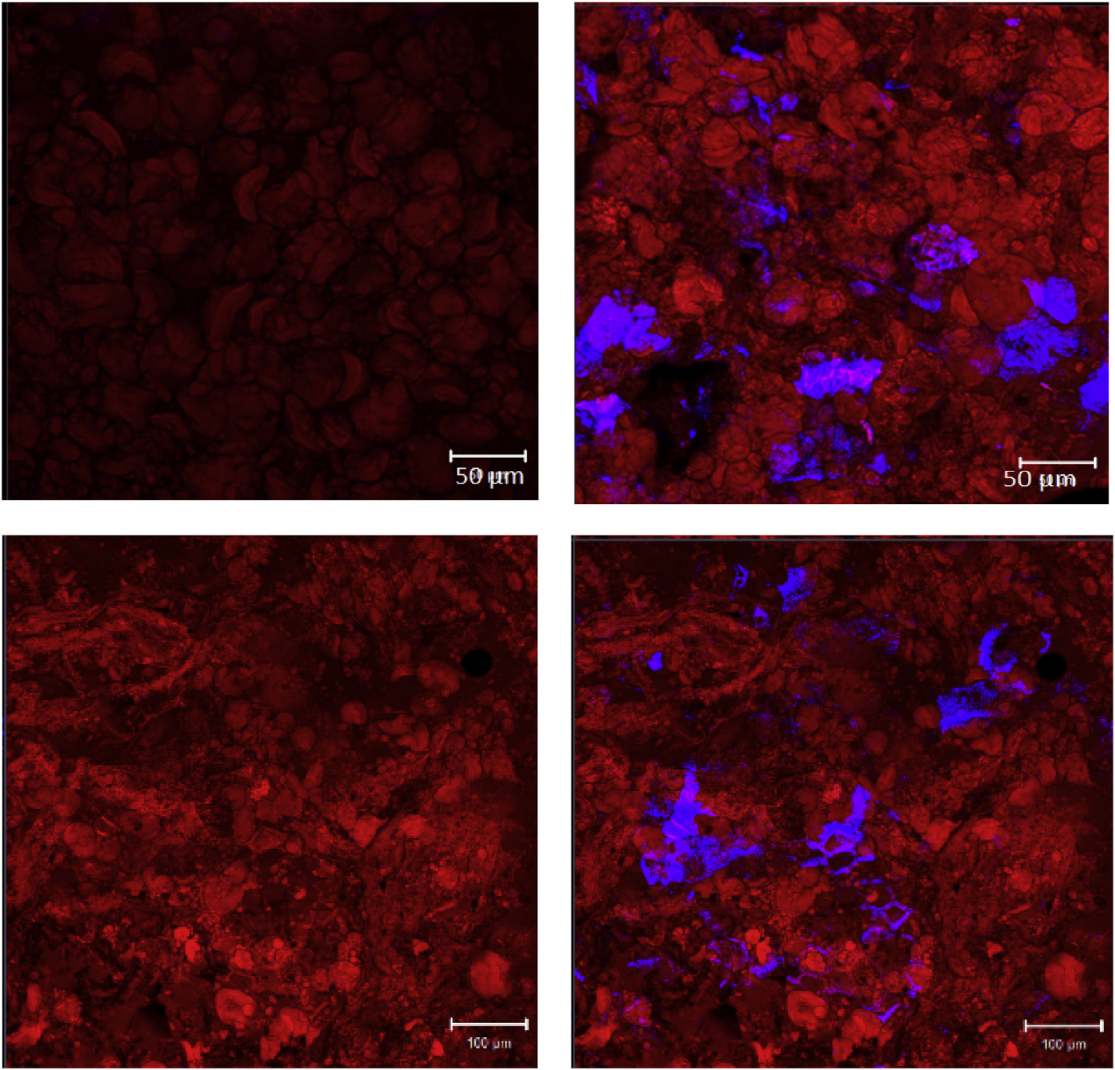
CLSM images of chapatti without (top left) and with bagasse fibers (top right and bottom).

**Fig. 3:**
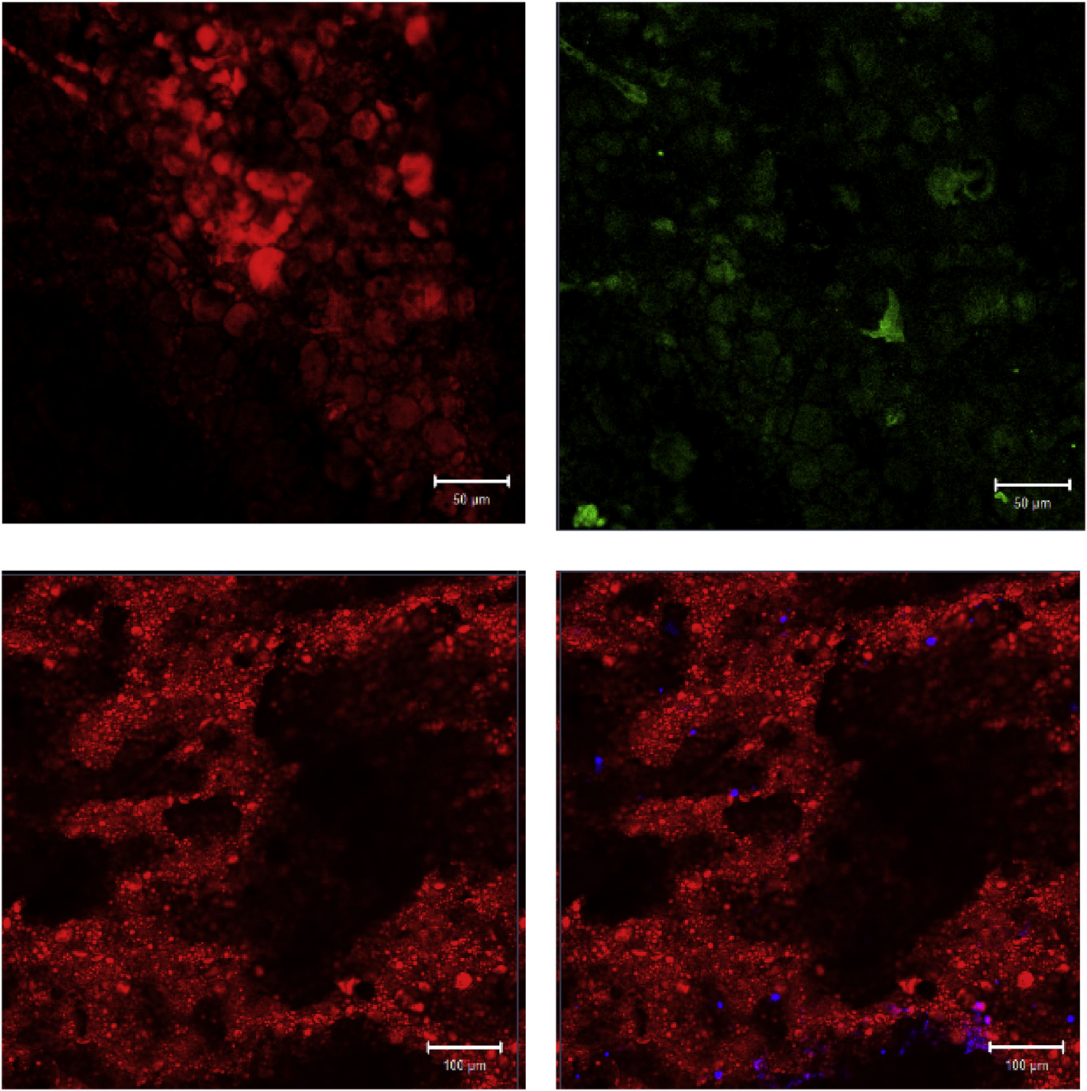
CLSM images of noodles without (top) and with sugar cane tops fibers (bottom).

The dataset of this article shows additional information about dispersion and integration of sugarcane bagasse (SCG) and sugarcane tops (SCT) fibers into flour to produce pasta (noodles) and bread (chapatti); furthermore, microbiological results, policosanol (PC) content and color measurement are reported. Fig. 1 displays photographs of noodles and chapatti prepared with 5 wt.-% SCT and 8 wt% SCB, respectively, and baked in microwave oven. Fig. 2 presents confocal laser scanning microscopy (CLSM) images of chapatti, before and after including SCB whereas Fig. 3 shows images of noodles, before and after the addition of SCT. For CLSM, chapatti samples were first coated with heparin and then imbued into rhodamine B (selective marker for carbohydrates, in red color) and calcofluor (selective marker for fibers, in blue color); noodles samples were only infused with calcofluor. Starch granules are detected as distorted spheres (in blue); SCB fibers are colored in red; both, SCB fibers and SCT are smoothly distributed into the products. In Table 1, the results of microbiological test are presented, according to the Mexican Standard NOM- 247-SSA1-2008.

**Table 1:** Microbiological analyses according to Mexican Standard NOM- 247-SSA1-2008.

Sample	Total coliforms UFC/g	Molds and yeasts UFC/g	Aerobic mesophiles, dilution 10^−4^ (UFC/g)
Pasta (Nodles) with SCT (sugar cane tops)	negative	<10 negative	5000
Fermented bread (Chapatti) with SCB (Sugar cane Bagasse)	negative	<10 UFC/g negative	2000
Permissible limit	<30	300	10,000

The policosanol (PC) content for SCB and SCTs fibers, with and without treatments, is shown in Table 2. PC compositions of the samples were identified by direct comparison of Individual standards: tricosanol (C_23_), tetracosanol (C_24_), heptacosanol (C_27_) and octacosanol (C_28_) [10].

**Table 2:** Policosanol analyses results.

Sample	Tricosanol, C_23_ (mg-mL^−1^)	Tetracosanol, C_24_ (mg-mL^−1^)	Heptacosanol, C_27_ (mg-mL^−1^)	Octacosanol, C_28_ (mg-mL^−1^)
SCT (control)	0.0096	0.0248	0.0118	0.6772
SCT (NaOH, MW)	0.0184	0.0012	0.0014	ND
SCT-MW	0.0387	0.0000	0.0013	ND
SCB (control)	0.0003	0.0038	0.0009	0.0054
SCB (NaOH, MW)	0.0006	0.0024	0.0037	0.0142
SCB-MW	0.0006	0.0022	0.0006	0.0058
Sugarcane epidermis	0.0035	0.0170	0.0066	0.2207

Color measurement was done using a color reader colorimeter and HunterLab colorimetric scale. The color index is determined on three parameters (equation (1)) and the results are shown in Table 3:

(1) $$IC=\left(a\right)\left(\frac{1000}{L.b}\right)$$

Where *a* is the red *versus* green scale, b is the yellow *versus* blue range and L is luminosity scale (0–100, dark *versus* light). Then, L value indicates the level of light or dark, *a* value redness or greenness, and b value yellowness or blueness. All three values are required to completely describe an object‘s color [11], [12], [13].

**Table 3:** Color Index by Hunterlab scale.

Sample	Color index
Fermented bread (control)	5.9
Fermented bread with SCB 5 wt-%	15.4
Fermented bread with SCB 8 wt-%	6.4
Pasta (control)	−2.9
Pasta with SCT 5 wt-%	31.2
Pasta with SCT 8 wt-%	--

## 2 Experimental design, materials, and methods

Noodles-type pasta and chapatti-type fermented bread were produced with traditional procedure, the SCB (8 wt%) and SCT (8 wt.-%) fibers were added during the mixing of dry ingredients; microwave oven (frequency of 2.45 GHz and power of 1100 W) and oven gas were used to bake these foods. The procedure to elaborate human food with the addition of SCB and SCT fibers is described in reference [1].

Confocal laser scanning microscopy (CLSM) study was carried out in Carl Zeiss LSM700 equipment (Carl Zeiss, Jena, Germany). Chapatti-type fermented bread samples were first protected with heparin and thereafter, both samples (pasta and fermented bread) were immersed into selective markers (rhodamine B for carbohydrates and calcofluor for fibers). Samples were rinsed to eliminate excess of markers.

Microbiological analyses were conducted according the Mexican Standard NOM- 247-SSA1-2008, for cereals, cereal flours, semolina, bread baking products; total coliforms, molds and yeasts and aerobic mesophiles were evaluated. 10 g of dry samples were mixed with 90 mL of nutrient solution followed of different logarithmic dilutions from 10^−1^ to 10^−6^. Thereafter, samples were storage in sterile bottle assays at 25 °C ± 1 during 120 h for the case of molds and yeasts evaluation, at 35 °C ± 2 during 48 h to analyze aerobic mesophiles, at 35 °C ± 2 for 24 h to evaluate total coliforms.

Policosanol analyses of fibers were performed by Gas chromatography using a chromatograph (GC-17A, Shimadzu, Japan) with Flame Ionization Detector, FID, automatic injector (AOC 20S) and capillary DB-5 column. Hydrogen was the carrier gas. An analytical micromill (Foss cyclotec™ 1093, Denmark) was used to grind the fibers. Rotary evaporator (Witeg Hahnvapor, Germany) was utilized to evaporate to dryness the ether extract under nitrogen. Fibers were prepared by grounding in analytical micromill at 40 mesh, some powders were MW treated and some were NaOH-MW treated; all the samples were hydrolyzed by 1.0 N NaOH in methanol refluxing, during 30 min; then, the mixtures were cooled down and filtered (Watman Nr. 42) using Büchner funnel vacuum filtration; HPLC grade water was added to the filtration system. After, for extraction, HPLC grade diethyl ether (Merck Index 14, 3806) was poured to the filtered solution; this step was performed three times with the same volume of diethyl ether; all the ether phases were collected into a separating funnel and washed with Millipore water [10]. Rotavap equipment was used to evaporate to dryness the ether extract under nitrogen (flasks were bubbled with nitrogen before connected them to the rotavap), at 26 °C and 30 rpm stirring; after, for removing the residual moisture, 5 g of anhydrous sodium sulfate (ACS grade, Fermont) was placed in the rotovap. The dried residues were washed with chloroform and transferred to volumetric flasks (5 mL), N-methyl-N-(trimethylsilyl) trifluoroacetamide (MSTFA, Sigma Aldrich) was used as derivatization reagent (500 μL) and chloroform was added to achieve 1 mL of sample. The systems were heated until up 60 °C during 20 min for derivatization. Finally, the analyses were conducted on a Gas chromatograph with FID, automatic injector and capillary DB-5 column, using a 1 mL sample volume each one. Hydrogen was the carrier gas (100 kPa), column temperature was maintained at 150 °C during 10 min; and then, a temperature ramp of 15 °C-min^−1^ was programmed to achieve 280 °C; injector and detector temperatures were 280 °C and 290 °C, respectively. Injection volume was 1 μL, split ratio was 1:25, and injection mode was “on column”.

Color index of fermented bread and pasta was determined with a colorimeter (Color Reader, CR-10 Plus, Konica Minolta, Japan) and HunterLab, scale, which is based on the Opponent-Color Theory. Measurement area was 78.5 mm^2^ (5 mm in diameter).
